# Examining domain‐specific cognitive associations with p‐tau217 in a cognitively normal sample: results from the BioFINDER‐Brown Study

**DOI:** 10.1002/alz.095290

**Published:** 2025-01-09

**Authors:** Hannah E Joyce, Alyssa N. De Vito, Phillip Caffery, Steve Salloway, Louisa I. Thompson

**Affiliations:** ^1^ Butler Hospital, Providence, RI USA; ^2^ Butler Hospital Memory and Aging Program, Providence, RI USA; ^3^ Alpert Medical School of Brown University, Providence, RI USA; ^4^ Brown University, Providence, RI USA

## Abstract

**Background:**

Emerging research on plasma Alzheimer’s disease (AD) biomarkers represents a new path towards earlier detection of AD pathology and potentially earlier intervention in cognitively normal individuals. Existing cognitive screening tools lack the sensitivity to distinguish between cognitively normal individuals with and without AD pathology. The present analysis investigated associations between performance on TabCAT digital assessment subtests and levels of plasma phosphorylated tau 217 (p‐tau217) in a cognitively healthy sample.

**Methods:**

Participants were cognitively normal (tMoCA ≥ 16, MMSE ≥ 26) adults (ages 50‐80) screening for the Biofinder‐Brown Study (n = 172, 68.6% female, mean_age_ = 64.3, mean_education_ = 17.2). Participants underwent a blood draw for p‐tau217 analysis. A p‐tau level of .17pg/ml was considered elevated. The first 58 individuals screened completed the full TabCAT Brain Health Assessment battery consisting of four subtests: Favorites (episodic memory), Match (processing speed), Line Orientation (visuospatial ability), and Animal Fluency (semantic fluency). Our screening protocol was then abbreviated, and the remaining 114 participants completed only the Favorites subtest. We used Spearman correlations to test associations between p‐tau levels and cognitive performance and independent samples t‐tests to examine differences in cognitive performance by p‐tau status (Elevated vs. Not).

**Results:**

Age‐, sex‐, and education‐adjusted TabCAT scores were not significantly associated with plasma p‐tau 217, except for animal fluency, which was negatively associated with p‐tau level (p = .029). TabCAT BHA Composite and MMSE also showed no associations with p‐tau level. None of the cognitive scores were able to distinguish between individuals with elevated and non‐ elevated plasma p‐tau. Individuals with higher education had significantly lower p‐tau (p = .05). There was no significant relationship between age and p‐tau level.

**Conclusions:**

Semantic fluency (animals subtest) performance was significantly correlated with p‐tau217, such that higher fluency scores were associated with lower p‐tau levels. Performances in other cognitive domains, including episodic memory, showed no significant associations. These results suggest that semantic fluency may be more sensitive to elevated p‐tau217 compared other cognitive abilities in cognitively healthy samples. However, the restricted range of cognitive performance in this highly educated sample may have reduced our ability to detect subtle associations between cognition and p‐tau217 levels.

